# UHPLC‐QTOF/MS‐based comparative metabolomics in pectoralis major of fast‐ and slow‐growing chickens at market ages

**DOI:** 10.1002/fsn3.2673

**Published:** 2021-12-02

**Authors:** Jian Zhang, Jing Cao, Ailian Geng, Haihong Wang, Qin Chu, Zhixun Yan, Xiaoyue Zhang, Yao Zhang, Huagui Liu

**Affiliations:** ^1^ Institute of Animal Husbandry and Veterinary medicine Beijing Academy of Agriculture and Forestry Sciences Beijing China

**Keywords:** Beijing‐You chicken, Cobb500 broiler, meat quality, pectoralis major, Untargeted metabolomics

## Abstract

The molecular regulatory mechanism underlying meat quality between different chicken genotypes remains elusive. This study aimed to identify the differences in metabolites and pathways in pectoralis major (breast muscle) between a commercial fast‐growing chicken genotype (Cobb500) and a slow‐growing Chinese native chicken genotype (Beijing‐You chickens, BYC) at market ages respectively based on ultra‐high‐performance liquid chromatography‐quadrupole/time of flight mass spectrometry (UHPLC‐QTOF/MS). Eighteen metabolites were identified as potential biomarkers between BYC and Cobb500 at market ages. Among them, L‐cysteine exhibited a higher relative intensity in BYC compared with Cobb500 and was enriched into 10 potential flavor‐associated KEGG pathways. In addition, the glycerophospholipid metabolism pathway was found to be associated with chicken meat flavor and the accumulation of sn‐glycerol 3‐phosphate and acetylcholine was more predominant in BYC than that in Cobb500, which were catalyzed by glycerophosphocholine phosphodiesterase (GPCPD1, EC:3.1.4.2), choline O‐acetyltransferase (CHAT, EC:2.3.1.6), and acetylcholinesterase (ACHE, EC:3.1.1.7). Overall, the present study provided some metabolites and pathways for further investigating the roles of the differences in meat flavor quality in breast muscle between Cobb500 and BYC at market ages.

## Graphical abstract

1. The glycerophospholipid metabolism pathway was associated with chicken meat flavor.
2. GPCPD1, CHAT, and ACHE were more active in BYC than in Cobb500.
3. L‐cysteine and glycerophospholipid metabolic pathways provide a different meat flavor.

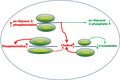

## 1 INTRODUCTION

During the past decades, breeding of commercial fast‐growing broilers has predominantly focused on increasing growth rate and improving body composition through classic quantitative genetic methods combined with modern molecular genetic technology (Berri et al., 2001; Cui et al., 2012), which has made it possible to slaughter the commercial fast‐growing broiler within 40 days of age (Petracci et al., 2015). Advances in genetics have dramatically increased the production of fast‐growing broilers. However, some adverse effects on the taste quality of the broiler meat have been noted (Velleman, 2019). For example, the fast‐growing broiler was found with worse meat quality, more degenerating muscle fibers, and higher plasma creatine kinase concentrations compared with the slow‐growing birds (Velleman, 2019; Wilson et al., 1990). Traditional, slow‐growing, yellow‐feathered chickens are preferred by many consumers (Huang et al., 2020; Jin et al., 2021; Rajkumar et al., 2016; Rizzi et al., 2007). For example, consumption of meat from domesticated slow‐growing yellow‐feathered chickens has increased to 6930 kilotons in 2019, an increase of 13.94% over that of 2018, and accounted for 41% of the total broiler meat marker in China (Xing et al., 2020).

Beijing‐You chicken (BYC) is a typical indigenous breed of China characterized by a slow growth rate, high‐quality traits of meat, and unique appearance (Chu et al., 2017). Recent studies have investigated the nutritional components, muscle fiber characteristics, color, texture, and structure of the meat of BYC, with the objective to characterize their special meat quality properties (Fu et al., 2015; Zhao et al., 2011). Moreover, the BYC has some distinct features suitable for serving consumers who prefer more flavor of chicken meat and more chewy texture (Zhao et al., 2012). In contrast, Cobb500 is a famous genetically improved commercial broiler with excellent growth rate, and meat yield, especially of breast muscle. Breast muscle yield could reach more than 20% of live weight after 28 d (www. Cobb‐Vantess.com), which is double that (around 10%) of BYC at similar live weight (Liu et al., 2013). In general, traditional slow‐growing chicken genotypes are considered to have superior quality compared with fast‐growing chicken genotypes (Li et al., 2009). Purchasing chicken according to appearance (plumage, skin, combs, etc.) is due to the lack of a clear understanding of the quality differences in different chicken genotypes at market ages. The biological processes and molecular pathways responsible for variations in meat quality in chicken species are still largely unknown. Especially, the accurate metabolomic analysis of muscle endogenous metabolites has scarcely been researched. Moreover, the underlying mechanisms and biomarkers related to the different meat qualities are still poorly defined. Therefore, a better understanding of these mechanisms would help improve the traits consistently and even identify biomarkers predictive of quality, which might open up new perspectives for chicken selection and production.

Metabolomics is a comprehensive analysis based on the whole metabolome, referring to the full complement of small molecule metabolites in organisms, tissues, or cells under well‐defined conditions (Patti et al., 2012). Over the past decades, metabolomics has also been well positioned both in livestock industry and in research, providing practical and economic advances, especially in livestock health, breeding, and production (Goldansaz et al., 2017). It has been applied in multiple fields, such as investigating the relationship between meat quality traits and muscle type (Muroya et al., 2014), predicting ultimate pH in chicken breast muscle (Beauclercq et al., 2016), identifying the metabolites in meat from livestock to evaluate meat quality (Dannenberger et al., 2017; Ueda et al., 2019), and identifying other important economic or breeding characteristics related to livestock. However, to date, only few studies have been performed on the metabolomic profiling of chicken in relation to the different chicken genotypes.

The main purpose of the present study was to identify the metabolic signatures and metabolic pathways resulting in meat quality differences between commercial fast‐growing chicken (Cobb500) and local slow‐growing chicken (BYC) at market ages respectively through an untargeted metabolomic technique. The potential metabolic biomarkers were also evaluated using validation data. This study provided insights into the underlying mechanisms associated with meat quality in different chicken genotypes.

## 2 MATERIALS AND METHODS

### 2.1 Reagents and materials

Liquid chromatography‐mass spectrometry (LC‐MS) grade water, HPLC grade methanol (MeOH), acetonitrile (ACN), and ammonium acetate were purchased from Honeywell (Muskegon, MI, USA). Ammonium hydroxide (NH_3_·H_2_O) was purchased from Sigma‐Aldrich (St. Louis, MO, USA).

### 2.2 Animals and tissue sampling

Forty‐eight 1‐day‐old female Cobb500 (Cobb‐Vantress) hatchlings (Beijing Poultry Breed Limited Company) and forty‐eight 1‐day‐old female local Chinese breed BYC birds (Institute of Animal Husbandry and Veterinary Medicine, Beijing Academy of Agriculture and Forestry Sciences) were commercially purchased. Individuals from both chicken genotypes had the same genetic backgrounds, and all birds entered the experiment simultaneously. Birds from both genotypes were randomly distributed into three replicate groups, with each group comprising 16 birds of BYC and Cobb500 birds, respectively. Birds were raised in an environmentally controlled room with six floor pens under the recommended environmental and nutritional conditions. Diets were made using the same material sources for the two breeds. All experiments were conducted according to the Guidelines for Experimental Animals established by the Ministry of Science and Technology (Beijing, China). Animal experiments were approved by the Science Research Department of the Institute of Animal Husbandry and Veterinary Medicine, Beijing Academy of Agriculture and Forestry Sciences (Beijing, China) (approval number: BAAFS‐IAHVM20181007).

Birds were slaughtered at market ages of 42 and 120 days for Cobb500 and BYC, respectively. Five birds with similar weight from each genotype of each group were first electrically stunned, and 15 birds were then killed by exsanguination for each genotype. Breast filets from both sides of the pectoralis major were used. Samples (200 mg per cube) from each left filet of every bird were snap‐frozen in liquid nitrogen prior to metabolomic profiling test, and the right filet of each bird was weighed and stored at 4℃ before the meat quality characteristic test, including intramuscular fat (IMF) and free amino acid (FAA). At last, for each breed, we randomly selected 10 samples for the metabolomic profiling test and 15 samples for the meat quality characteristic test.

### 2.3 Meat quality characteristics

Initial muscle pH_15_ (i.e., the pH value at 15 min postmortem) and pH_u_ (i.e., the pH value at 24 h postmortem) were measured in a chilling room with a pH‐STAR CPU meter (Matthäus, Germany). Drip loss and cooking loss were measured as previously described (Hofmann & Schieberle, 1995). The Warner–Bratzler shear force (WBSF) analysis was performed as described earlier (Zhao et al., 2011). Shear force measurements were conducted on a TMS‐PRO (FTC Co.) equipped with a WBSF head at a crosshead speed of 200 mm/min. The shear force of each breast muscle sample was defined as the arithmetic mean value of six cuts from the same filet. IMF content of breast muscle was determined using the petroleum ether percentage in the muscle (dry weight), and petroleum ether was measured using a Soxhlet apparatus (Lee et al., 2004). An amino acid analyzer (L‐8900, HITACHI) was used to obtain the value of FAA following the method described by Li et al. (2017) with a slight modification. All analyses were carried out in duplicate.

### 2.4 Sample preparation of metabolomics study

Sample preparation was carried out as described by Lu and Xu (2017) with a slight modification. Breast muscle samples were weighed before metabolite extraction. Dried lyophilized samples were ground in a 2‐mL Eppendorf tube that contained a 5‐mm tungsten bead for 1 min at 65 Hz in a grinding mill. First, metabolites were extracted using 1 ml precooled mixtures of methanol, acetonitrile, and water (v/v/v, 2:2:1), then ultrasonicated at 40 kHz for 1 h, and shaken in ice baths. Subsequently, the mixture was placed at −20°C for 1 h and centrifuged at 14,000 × *g* for 20 min at 4°C. The supernatants were recovered and concentrated to dryness using vacuum. Moreover, to insure data quality for metabolic profiling, quality control (QC) samples were prepared by pooling aliquots of all samples, preparing for data normalization. Following the same procedure used for the experiment samples, QC samples were prepared and analyzed. Acetonitrile (50%) was used for dissolving dried extracts. After being filtered with disposable 0.22 µm cellulose acetate, each sample was transferred into 2‐ml HPLC vials and stored at −80°C for downstream analysis.

### 2.5 UHPLC‐MS/MS analysis

We used the UHPLC‐QTOF‐MS system (UHPLC, 1290 Infinity LC, Agilent Technologies) and TripleTOF 5600 (AB Sciex) for the metabolomic profiling analysis as previously described (Plumb et al., 2005). A 2.1 ×100 mm ACQUIY UPLC BEH 1.7 μm column (Waters, Ireland) was used to prepare the samples for hydrophilic interaction liquid chromatography (HILIC) separation with a flow rate of 0.5 ml/min. Two mobile phases were selected for HILIC chromatography: 25 mM ammonium acetate and 25 mM ammonium hydroxide in water for A and acetonitrile (ACN) for B. Positive mode (ESI+) and negative mode (ESI‐) were used to acquire MS data. For MS acquisition, the m/z range was set from 60 to 1200 Da. The accumulation time was set at 0.15 s/spectra for TOF MS scanning. For MS/MS acquisition, the m/z range was set from 25 to 1200 Da, and the accumulation time was set at 0.03 s/spectra for product‐ion scanning. The production scan was performed with information acquired in the selected high‐sensitivity mode. We set the collisional energy at 30 V with ±15 eV and declustering potential as ±60 V. Six blank (75% ACN in water) and QC samples were injected during acquisition.

### 2.6 Multivariate statistical analysis

ProteoWizard (Chambers et al., 2012) was used to convert the raw MS data (.wiff) to the MzXML format. Feature detection, alignment, and retention time correction were processed using the R package XCMS (version 3.2). Metabolites were identified with an accuracy mass (<25 ppm) and standardized MS/MS data. We only kept extracted‐ion features with more than 50% of the nonzero values in at least one group. Then, all modeling and multivariate data analyses were performed using the SIMCAP software (version 14.0, Umetrics AB, Umeå, Sweden). The data were scaled with Pareto scaling. We built models on PCA, partial least‐square discriminant analysis (PLS‐DA), and orthogonal partial least‐square discriminant analysis (OPLS‐DA). OPLS‐DA could indicate discriminating metabolites with variable importance on projection (VIP). Statistically significant metabolites were defined as metabolites with VIP values higher than 1.0 and *p‐*values <.05. Fold change was the logarithm transformation of the average mass response (area) ratio between the two classes. Cluster analyses were performed with the identified differential metabolites using the R package.

### 2.7 KEGG enrichment analysis

To identify the significant biological pathways, KEGG pathway analysis was performed with the differential metabolite data using the KEGG database (https://www.kegg.jp/). The KEGG enrichment analysis was carried out with Fisher's exact test, followed by false discovery rate (FDR) correction for multiple testing. Only KEGG pathways with FDR < 0.05 were considered significant.

### 2.8 Statistical analysis

The parameters were shown as averages and standard deviations. Analysis of data on carcass and muscle characteristics was performed using one‐way ANOVA in the GLM procedure of SAS version 9.2 (SAS Institute Inc.). Statistical significance was set at *p* < .05.

## 3 RESULTS

### 3.1 Breast muscle quality was distinct between the BYC and Cobb500 broilers

We measured the meat quality characteristics of both chicken genotypes through the postmortem examination of each sample (Table 1). We found that the live weight, breast weight, pH_15_, Warner–Bratzler shear force, drip loss, and cook loss of BYC were significantly lower (*p* < .05) than those of Cobb500. The pH_u_ from both genotypes was in the normal range (5.7–6.1) and exhibited no significant difference (*p* > .05). As expected, the content of IMF and dry matter (DM) in BYC were significantly higher (*p* < .05) than that in Cobb500 broiler. Regarding the FAA concentrations, 17 out of 18 showed a significant difference (*p* < .05) between the two chicken genotypes, except for L‐carnosine (*p* > .05). Only L‐anserine was significantly higher in BYC than in Cobb500.

**TABLE 1:** Growth and meat quality characteristics of breast muscle between Cobb500 and Beijing‐you chicken (BYC) at market ages (*n* = 15)

Items	BYC	Cobb 500	*p*‐value
Live weight (g)	1205 ± 136	2591 ± 169	<0.0001
Breast weight (g)	66.85 ± 8.05	284.79 ± 17.27^a^	<0.0001
pH_15_	6.21 ± 0.37	6.51 ± 0.19	0.008
pH_u_	5.90 ± 0.10	5.98 ± 0.15	0.0762
WBSF (*N*)	29.15 ± 8.46	48.53 ± 11.59	<0.0001
Drip loss (%)	2.83 ± 0.78	9.35 ± 3.28	<0.0001
Cook loss (%)	14.40 ± 3.29	19.48 ± 3.69	<0.0001
Phosphoserine (P‐Ser, g/10 kg)	0.31 ± 0.06	0.46 ± 0.08	<0.0001
Taurine (Tau, g/10 kg)	2.92 ± 0.51	6.12 ± 1.47	<0.0001
L‐aspartic acid (Asp, g/10 kg)	0.41 ± 0.15	0.75 ± 0.31	0.0007
L‐threonine (Thr, g/10 kg)	31.50 ± 16.36	56.51 ± 17.69	0.0004
L‐serine (Ser, g/10 kg)	1.65 ± 0.49	4.50 ± 1.45	<0.0001
L‐glutamic acid (Glu, g/10 kg)	3.36 ± 1.48	10.98 ± 3.73	<0.0001
Glycine (Gly, g/10 kg)	1.62 ± 0.34	5.40 ± 1.64	<0.0001
L‐alanine (Ala, g/10 kg)	4.38 ± 0.81	10.20 ± 2.37	<0.0001
L‐valine (Val, g/10 kg)	0.78 ± 0.27	1.94 ± 0.50	<0.0001
L‐isoleucine (Ile, g/10 kg)	0.44 ± 0.20	1.09 ± 0.30	<0.0001
L‐leucine (Leu, g/10 kg)	0.98 ± 0.40	2.09 ± 0.52	<0.0001
L‐tyrosine (Tyr, g/10 kg)	0.70 ± 0.25	1.83 ± 0.48	<0.0001
L‐phenylalanine (Phe, g/10 kg)	0.67 ± 0.22	1.46 ± 0.67	0.0002
β‐Alanine (β‐Ala, g/10 kg)	1.97 ± 0.79	9.23 ± 3.90	<0.0001
L‐lysine (Lys, g/10 kg)	1.44 ± 0.38	4.40 ± 1.64	<0.0001
L‐anserine (Ans, g/10 kg)	339.36 ± 23.71	221.82 ± 44.39	<0.0001
L‐carnosine (Car, g/10 kg)	84.00 ± 17.57	94.05 ± 24.37	0.2055
L‐arginine (Arg, g/10 kg)	1.12 ± 0.38	5.59 ± 2.54	<0.0001
IMF (%)	4.68 ± 2.88	2.97 ± 0.76	0.0338
DM (%)	26.84 ± 0.75	24.36 ± 0.70	<0.0001

Note

Means within same rows followed by a different superscript were significantly different (*p* < .05). pH_15_ and pHu, the pH value measured at 15 min or 24 h postmortem, respectively; WBSF, Warner–Bratzler shear force; IMF, intramuscular fat content of breast muscle; DM, dry matter content of breast muscle; BYC and Cobb500 in the table represent Beijing‐You Chicken and Cobb500, respectively.

### 3.2 Breast muscle metabolic profiles significantly differed between BYC and Cobb500

**FIGURE 1:**
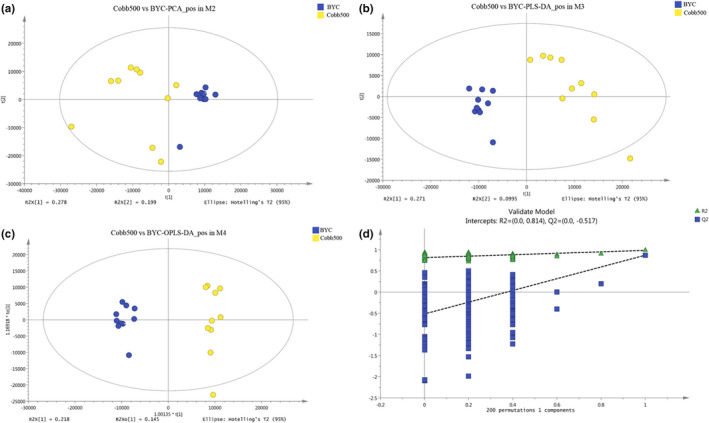
Significant separation of overall metabolic file for breast muscle extracts in positive ionization mode (ESI+) between the two chicken genotypes. a–d, indicate the separation using score plots of PCA, the separation using PLS‐DA, the separation using OPLS‐DA, and the model validation using a permutation test with 200 times, respectively. BYC and Cobb500 in the plot represent Beijing‐You Chicken and Cobb500 samples, respectively

**FIGURE 2:**
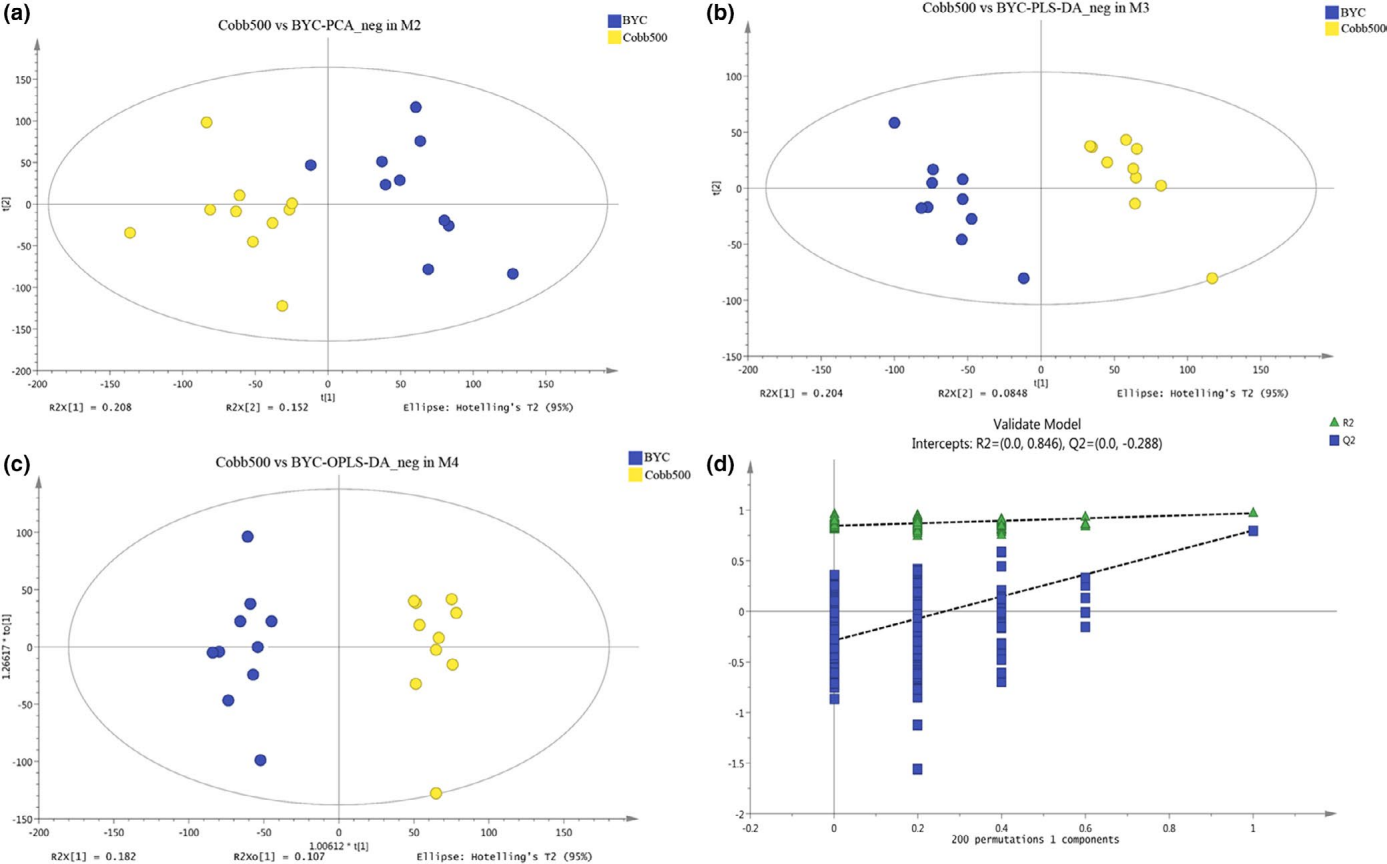
Significant separation of overall metabolic file for breast muscle extracts in negative ionization mode (ESI‐) between the two chicken genotypes. a–d, indicate the separation using score plots of PCA, the separation using PLS‐DA, the separation using OPLS‐DA, and the model validation using a permutation test with 200 times, respectively. BYC and Cobb500 in the plot represent Beijing‐You Chicken and Cobb500 samples, respectively

To determine which metabolites were associated with overall physiological differences between the two chicken genotypes, we selected the 19,419 ESI+ and 28,942 ESI− features for subsequent analysis. Concerning the 19,419 ESI+ features, principal component analysis (PCA) with the metabolic profiles showed a clear separation (Figure 1a), indicating the different breast muscle metabolic compositions of the two chicken genotypes. We performed another two analyses, using different algorithms with PCA: partial least‐squares discriminant analysis (PLS‐DA, R^2^Y = 0.969, Q^2^Y = 0.784) (Figure 1b) and orthogonal partial least‐squares discriminant analysis (OPLS‐DA, R^2^Y = 0.969, Q^2^Y = 0.799) (Figure 1c), which further confirmed the significant differences in the metabolic profiles. Our permutation test (*n* = 200) in Figure 1d, with the negative intercept of the Q^2^ regression line and all permuted R^2^‐values lower than the original point, further validated the reliability of PLS‐DA and OPLS‐DA. Consistent with the ESI+ features, the same analyses of the ESI‐ features brought similar significant separation with the permutation test (Figure 2a–d).

### 3.3 Identification of 18 potential metabolite biomarkers for both breeds

With VIP threshold and *p*‐value of two‐tailed Student's *t* test set to 1.0 and 0.05, we obtained 100 and 88 discriminating metabolites from BYC and Cobb500 in negative and positive ion modes, respectively (Tables S1 and S2). In this study, comprehensive multivariate data analysis showed significant differences in the concentration of creatine, taurine, amino acids, organic acids, and peptides. To intuitively inspect the variation in the relative intensity of metabolite concentrations between these two different chicken genotypes, we performed heat map and hierarchical clustering analysis (HCA) according to the relative intensity of the differential metabolites (171 de‐redundancy metabolites) (Figure S1).

**FIGURE 3:**
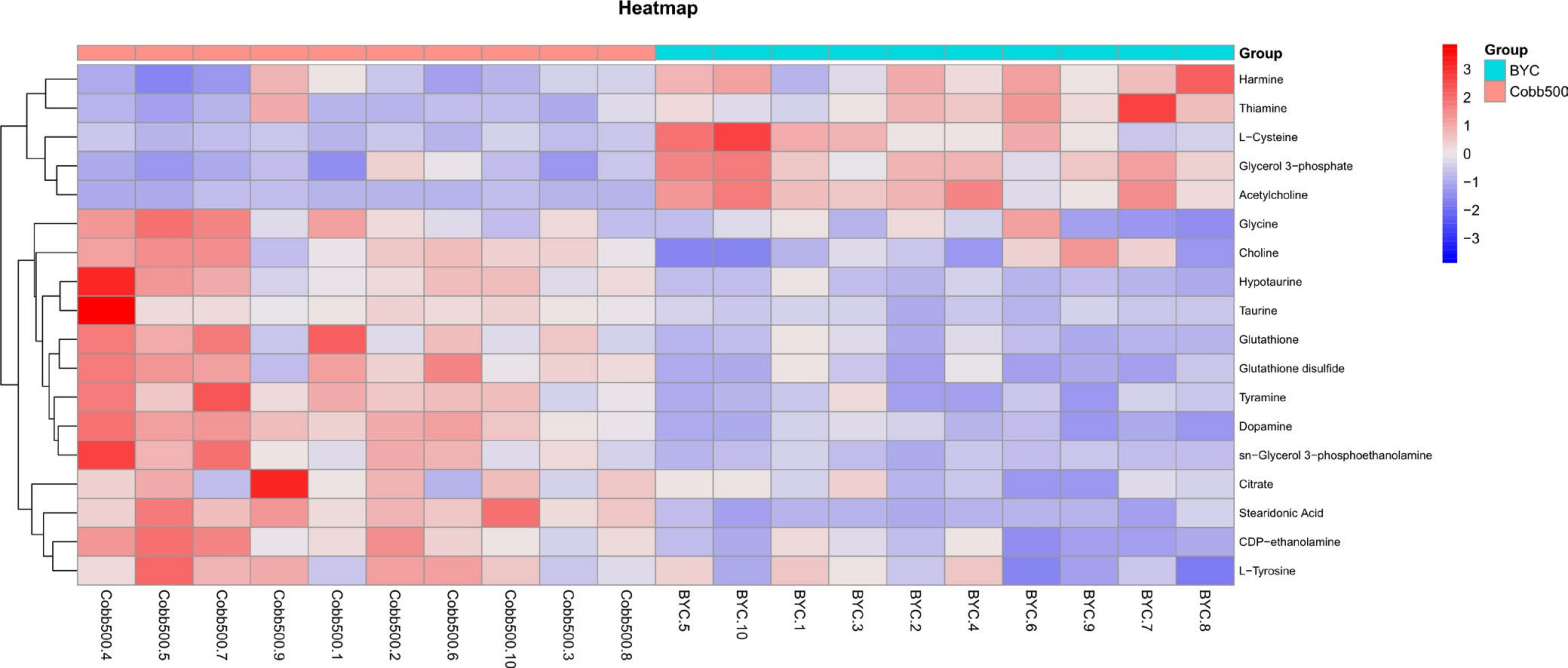
Heat map of the 18 differential metabolites responding to breast muscle at market ages of BYC and Cobb500. Each row represents a metabolite feature, and each column represents a sample. BYC and Cobb500 in the plot represent Beijing‐You Chicken and Cobb500 samples, respectively

Based on the results of the HCA, 18 metabolites were ascertained as potential biomarkers to discriminate Cobb500 from BYC (Table 2). The relative standard deviation (RSD) of the 18 metabolites varied from 3.70% to 21.71%, with a median of 10.72%, indicating the robustness of the metabolomic platform. As shown in Figure 3, the heat map showed that the content of 13 metabolites increased and that of 5 metabolites decreased in Cobb500 compared with BYC, and the box plots of peak intensities of the most intense mass peak from each metabolite demonstrated clear differential metabolic profiles between BYC and Cobb500 at market ages, with the fold change values ranging from 0.19 to 3.41 in Cobb500 versus BYC (Figure S2; Table 2). Briefly, acetylcholine, L‐cysteine, glycerol 3‐phosphate, thiamine, and harmine exhibited lower relative intensities in Cobb500 compared with BYC. In comparison, higher relative intensities were observed for L‐tyrosine, glycine, choline, tyramine, dopamine, CDP‐ethanolamine, citrate, taurine, glutathione, sn‐glycerol 3‐phosphoethanolamine, glutathione disulfide, hypotaurine, and stearidonic acid in Cobb500 compared with BYC.

**TABLE 2:** Metabolomic biomarkers used to discriminate Cobb500 from Beijing‐You chicken (BYC, *n* = 10)

Metabolite	*m*/*z*	RT(s)	VIP	*p*‐value	FC	ESI	Direction	RSD %
Acetylcholine	146.11	192.75	1.74	5.43376E−07	0.19	POS	Down	9.42
Choline	104.10	148.85	2.33	0.012224334	1.43	POS	Up	16.12
sn‐Glycerol 3‐phosphoethanolamine	216.06	383.04	1.32	0.00050835	2.24	POS	Up	12.51
sn‐Glycerol 3‐phosphate	171.01	411.24	10.69	4.09704E−05	0.48	NEG	Down	11.01
CDP‐ethanolamine	445.06	423.93	1.46	0.000293558	1.59	NEG	Up	8.02
Thiamine	326.14	324.02	1.13	0.001807871	0.54	POS	Down	14.40
L‐tyrosine	180.07	280.70	4.41	0.010693002	1.30	NEG	Up	3.95
Glycine	74.03	341.81	1.51	0.030143944	1.33	NEG	Up	9.55
L‐cysteine	137.04	142.21	1.09	0.000666615	0.35	NEG	Down	6.49
Glutathione	308.09	478.58	2.63	0.00114245	2.13	POS	Up	19.74
Glutathione disulfide	613.16	480.63	9.02	6.90169E−05	2.78	POS	Up	6.63
Taurine	126.02	285.70	6.89	0.00959586	2.08	POS	Up	3.70
Hypotaurine	108.01	322.14	1.01	0.001112739	2.89	NEG	Up	13.96
Harmine	271.11	378.58	1.74	0.002693309	0.60	NEG	Down	5.85
Dopamine	136.07	296.83	1.78	1.12556E−06	1.53	POS	Up	7.58
Tyramine	120.08	278.40	2.18	9.24928E−05	1.48	POS	Up	11.85
Citrate	191.02	463.42	6.30	0.027347706	1.79	NEG	Up	21.71
Stearidonic acid	275.21	42.65	2.41	1.34304E−07	3.41	NEG	Up	10.45

*Abbreviations*: FC, fold change; RT, retention time; VIP, variable importance in the projection.

*Note:* *m/z* of ion used for univariate analysis; POS, ESI+; NEG, ESI−; Direction “Up” indicates a relative high concentration present in Cobb500 samples, and FC (fold change) was calculated from the ratio of the mean values of Cobb500 samples relative to Beijing‐You chicken (BYC) samples, while “Down” means a relative low concentration compared with the BYC samples, and FC was calculated from the ratio of the mean values of Cobb500 relative to BYC; RSD % = relative standard deviation for QC samples.

### 3.4 The L‐cysteine and glycerophospholipid metabolic pathways are potentially associated with breast muscle quality

**FIGURE 4:**
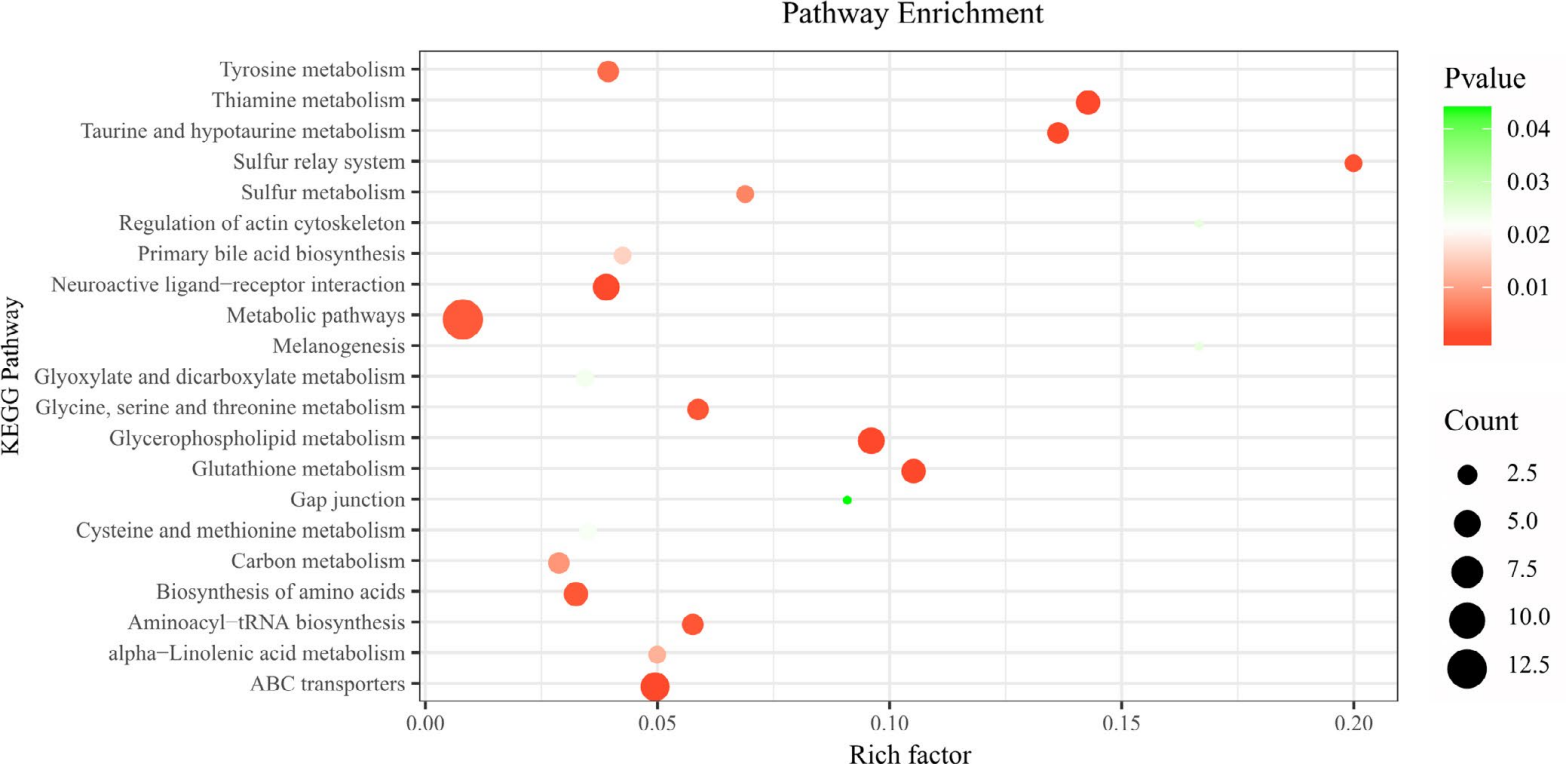
Metabolic pathway enrichment analysis of differential metabolites in breast muscle between BYC and Cobb500. All matched pathways are displayed as circles; the color and size of each circle are based on the *p*‐value and the pathway impact value, respectively. Redder and bigger circus indicates lower *p*‐values and greater count numbers, respectively

To reveal the significantly different pathways in the breast muscle of the two chicken genotypes, we further performed enrichment analysis and pathway topology analysis based on the 18 important metabolites listed in Table 2. Sixteen KEGG pathways were identified with adjusted *p*‐values <.05, indicating an association with chicken breast muscle quality. Among these 16 KEGG pathways, the glycerophospholipid metabolism pathway was the most enriched (Figure 4; Table S3). Actin cytoskeleton and sulfur relay system pathways were only enriched by BYC‐rich metabolites. In contrast, the tyrosine metabolic pathway and primary bile acid biosynthesis were only enriched by Cobb500‐rich metabolites (Table S3). In addition, the other 12 pathways enriched by metabolites significantly increased in both chicken genotypes (Table S3). L‐cysteine, which was significantly increased in BYC, was enriched in 10 pathways (thiamine metabolism, glutathione metabolism, taurine and hypotaurine metabolism, ferroptosis, sulfur relay system, glycine, serine and threonine metabolism, aminoacyl‐tRNA biosynthesis, biosynthesis of amino acids, sulfur metabolism, and carbon metabolism), indicating that L‐cysteine might play a key role in the development of meat flavor.

Among the five metabolites enriched in the glycerophospholipid metabolism pathway, sn‐glycerol 3‐phosphate (glycerol 3‐phosphate) and acetylcholine were significant metabolites for BYC, with 0.48‐ and 0.19‐fold, respectively, compared with Cobb500 (fold = Cobb500/BYC) (Table S3). Sn‐glycero‐3‐phosphocholine, phosphocholine, and choline were significantly increased metabolites in Cobb500 with 7.02‐, 2.37‐, and 1.43‐fold, respectively, compared with BYC (fold = Cobb500/BYC) (Table S2).

## 4 DISCUSSION

The extreme focus on increasing growth rate and muscle yield in commercial fast‐growing chickens has not taken into account the quality of taste. To provide a higher degree of selectivity and sensitivity for identification of metabolites contributing to the various taste qualities in different chicken genotypes at market ages, we combined UHPLC separation and QTOF/MS detection to identify meat quality‐related signatures and pathways of metabolism. We provide a reference for chicken breeding and bioenhancement through gaining insight into the differences in meat quality‐associated metabolism between two different chicken genotypes (BYC and Cobb500) at market ages.

Commercial fast‐growing chicken genotypes usually have higher live and carcass weights, as well as breast and thigh‐drumstick weights, compared with slow‐growing chicken genotypes (Cömert et al., 2016), which were in line with our observations. We measured several meat quality‐associated characteristics and found that the live weight and breast weight of Cobb500 were significantly higher than that of BYC (*p* < .05). In addition, we observed that drip loss and cook loss were significantly lower in BYC than that in Cobb500. Drip loss and cook loss reflect muscle water‐holding capacity (Ab Aziz et al., 2020). Additionally, drip loss has a negative association with color parameters (lightness and redness) (Gotardo et al., 2015). Our results showed that drip loss and cook loss were significantly lower (*p* < .05) in BYC breast muscle than that in Cobb500 broilers, indicating a better water‐holding capacity, lightness, and redness in the case of BYC. pH_15_ was significantly lower in BYC than that in Cobb500, while no significant differences in pH_u_ were found between the two chicken genotypes. Postmortem pH is usually thought to be a key meat quality factor (EI Rammouz et al., 2004). Since a low pH might increase the risk of producing white meat with reduced water retention, the lower postmortem pH might suggest a worse meat quality (Dransfield & Sosnicki, 1999). The final pH usually affects the texture, color, and water‐holding capacity of both raw and cooked meat, and an early pH drop is mainly associated with drip loss and lightness of raw meat (Le Bihan‐Duval et al., 2008). The lower pH_15_ in BYC compared with Cobb500 might be due to stress‐ and behavioral activity‐related differences between the two chicken genotypes. Stress response, behavioral activity, and muscle glycogen levels during or prior to slaughter on a shackle line were reportedly involved in the alterations of postmortem pH_15_ and pH_u_ in general (Berri et al., 2005; Debut et al., 2005). Considering the equal pH_u_ range and the significantly lower drip loss and cooking loss compared with Cobb500, indicating a better water‐holding capacity in BYC, the negative effect of the lower BYC pH_15_ might not be considerable. The Warner–Bratzler shear force is an indicator of texture tenderness. In our study, we found that the slow‐growing chicken genotype displayed significantly lower shear force than the commercial fast‐growing chicken genotype, indicating a more tender texture. IMF has a beneficial effect on the succulency, tenderness, and palatability of meat and is therefore a main meat quality‐determining factor in multiple countries (Cui et al., 2018; Nishimura et al., 1999). We found that IMF and DM were significantly higher in BYC, indicating better meat quality, which is in good agreement with published studies (Liu et al., 2019).

Among the 18 important FAAs obtained in this study, only anserine was significantly higher in BYC. Anserine is the major histidine‐containing dipeptide (HCD) in poultry meat (Jung et al., 2013). HCD plays a variety of physiological roles, including intracellular buffering and free radical quenching in skeletal muscle, which not only participates in the direct quenching mechanism of reactive carbonyl substances but also prevents the formation of advanced glycoxidation and advanced lipoxidation (Aldini et al., 2005; Boldyrev et al., 2013). This suggested that consumption of BYC might elicit health benefits. The other 17 FAAs were significantly increased in Cobb500, and L‐glutamic acid (Glu) concentration was approximately threefold higher than that in BYC, which were in good agreement with the higher levels of amino acid metabolites of Cobb500 compared with that of BYC. Shinobu et al. (1995) concluded that Glu greatly contributed to meat taste when investigating the taste‐active components with synthetic meat extracts using addition and omission tests. Thus, Glu was considered the most important component in meat, affecting the taste of brothy and umami (Imanari et al., 2008). The higher concentration of Glu in Cobb500 than in BYC is the reason for better taste in the former.

To gain further insight into the mechanism involved in the differences in meat quality characteristics, we obtained the metabolic profiles of both chicken genotypes. Several methods, including PCA, PLS‐DA, and OPLS‐DA, confirmed the significantly different compositions of metabolites in both chicken genotypes. Using statistical analysis, five potential metabolites (acetylcholine, L‐cysteine, glycerol 3‐phosphate, thiamine, and harmine) for BYC breast meat and 13 potential metabolites (L‐tyrosine, glycine, choline, tyramine, dopamine, CDP‐ethanolamine, citrate, taurine, glutathione, sn‐glycerol 3‐phosphoethanolamine, glutathione disulfide, hypotaurine, and stearidonic acid) for Cobb500 breast muscle were obtained. Among the five potential metabolites for BYC breast muscle, L‐cysteine and thiamine have been reported to be associated with meat flavor. When L‐cysteine reacts with sugar, it imparts a special taste to meat, especially chicken and pork (Li et al., 2017). The flavor of food is generally determined by volatiles from the Maillard reaction (Cerny, 2008). In the Maillard reaction system, L‐cysteine, D‐xylose, and thiamine are important ingredients for meat‐like flavors, which play important roles as meat aroma precursors, especially for sulfur‐containing odorants (Mottram, 1998). Sulfur aroma substances often play a dominant role in meat flavor and are considered the most powerful aroma substances (Cerny, 2008). BYC breast meat gained more meat flavor because of the presence of L‐cysteine and thiamine. Taurine is different from other amino acids as it is barely incorporated into proteins. It has also been reported that taurine could enhance the growth performance of broilers (MacLeod & Seyyedain‐Ardebili, 1981). Although the role of taurine in chicken remains unclear, the present findings clearly showed that taurine content of Cobb500 breast was 2.08‐fold higher than that of BYC. This might provide further evidence that taurine was associated with growth performance.

**FIGURE 5:**
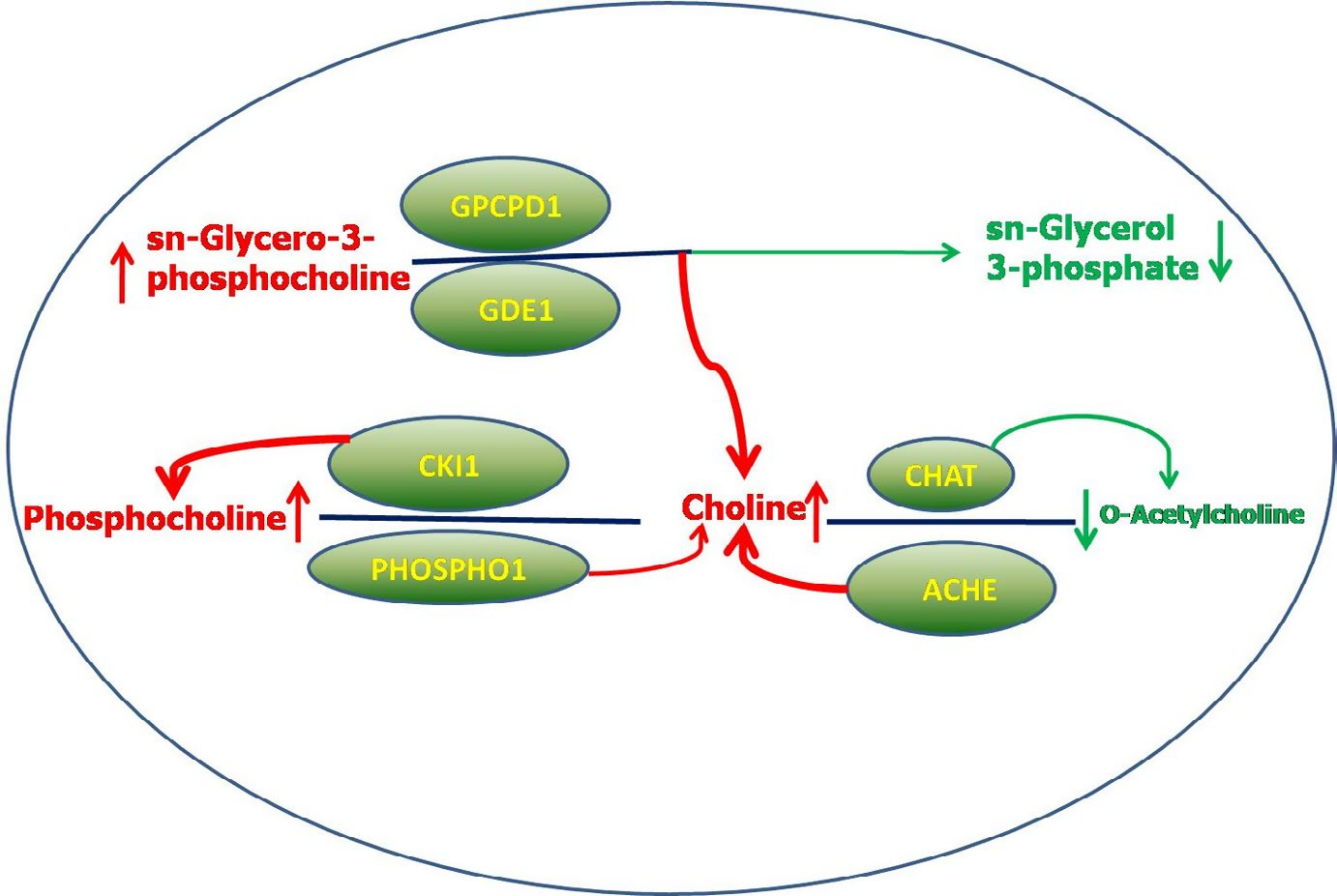
Glycerophospholipid metabolic pathway networks. The relative intensities of metabolites in Cobb500 compared with those of BYC are marked in red (upregulated, Cobb500 versus BYC) and green (down‐regulated, Cobb500 versus BYC), respectively

Of note in the KEGG pathway enrichment analysis is the glycerophospholipid metabolism pathway. As the major lipid components in all biological membranes, glycerophospholipids play crucial roles in both the structure and function of all animal cells, forming cell membranes and mediating signal transduction (Werkhoff et al., 1990). The sn‐glycero‐3‐phosphocholine could be catalyzed to sn‐glycerol 3‐phosphate by glycerophosphocholine phosphodiesterase (GPCPD1, EC:3.1.4.2). The significantly higher concentration of sn‐glycerol 3‐phosphate in BYC indicated a higher GPCPD1 activity in BYC compared with Cobb500. As the acetylcholine and choline accumulation in Cobb500 were 0.18‐ and 1.43‐fold relative to those in BYC, the acetylcholine‐to‐choline transition is catalyzed by acetylcholinesterase (ACHE, EC:3.1.1.7). In contrast, the choline‐to‐acetylcholine transition is catalyzed by choline O‐acetyltransferase (CHAT, EC:2.3.1.6). These results suggest that CHAT was more active in BYC and so did ACHE in Cobb500. In short, sn‐glycero‐3‐phosphocholine and choline progression toward the generation of sn‐glycerol 3‐phosphate and acetylcholine were more active in BYC than in Cobb500 (Figure 5). The difference in the glycerophospholipid metabolic pathway might provide an explanation for the significant IMF difference between the two chicken genotypes. Moreover, considering that choline could decrease triglycerides, very low‐density lipoprotein (VLDL), low‐density lipoprotein (LDL), and serum total cholesterol (Rahnama et al., 2020), the higher concentration of choline could be assumed to be responsible for the lower IMF in Cobb500.

## 5 CONCLUSION

In summary, the present study provided 18 potential metabolite biomarkers between the breast muscle of BYC and Cobb500 at market ages and suggested metabolic pathways of L‐cysteine and glycerophospholipid were potentially associated with breast meat quality. These findings could potentially provide new insights into the physiological and molecular mechanisms of meat flavor quality, as well as resources for biomarker identification to improve these traits.

## CONFLICT OF INTEREST

The authors declare that they have no conflict of interest.

## AUTHOR CONTRIBUTIONS

**Jian Zhang:** Conceptualization (equal); Writing‐original draft (lead). **Jing Cao:** Investigation (equal). **Ailian Geng:** Investigation (equal). **Haihong Wang:** Investigation (equal). **Qin Chu:** Data curation (equal). **Zhixun Yan:** Data curation (equal). **Xiaoyue Zhang:** Resources (equal). **Yao Zhang:** Resources (equal). **Huagui Liu:** Conceptualization (equal); Writing‐review & editing (equal).

## ETHICAL APPROVAL

All experiments were conducted according to the Guidelines for Experimental Animals established by the Ministry of Science and Technology (Beijing, China). Animal experiments were approved by the Science Research Department of the Institute of Animal Husbandry and Veterinary Medicine, Beijing Academy of Agriculture and Forestry Sciences (Beijing, China) (approval number: BAAFS‐IAHVM20181007).

## Supporting information

Figure S1

Figure S2

Table S1

Table S2

Table S3

## Data Availability

The data used to support the findings of this study are available from the corresponding author upon request.
