# Supplementary figures and images for: UHPLC‐QTOF/MS‐based comparative metabolomics in pectoralis major of fast‐ and slow‐growing chickens at market ages
Source: Food Sci Nutr. 2021 Dec 2;10(2):487–98. doi: 10.1002/fsn3.2673 (PMC8825714; doi:10.1002/fsn3.2673)

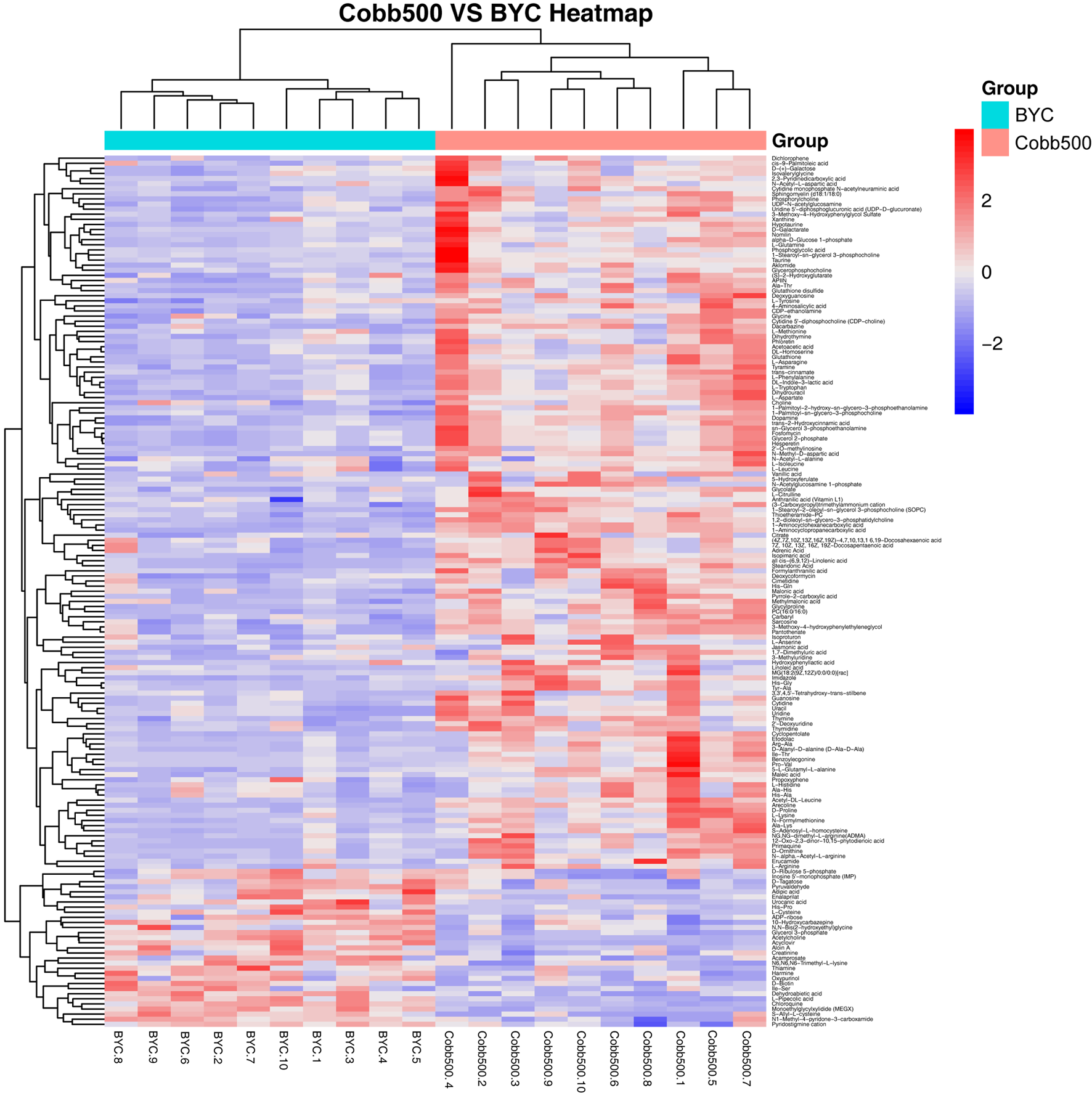

Supplement: Supplementary file 1 — Figure S1 [file FSN3-10-487-s004.tif]

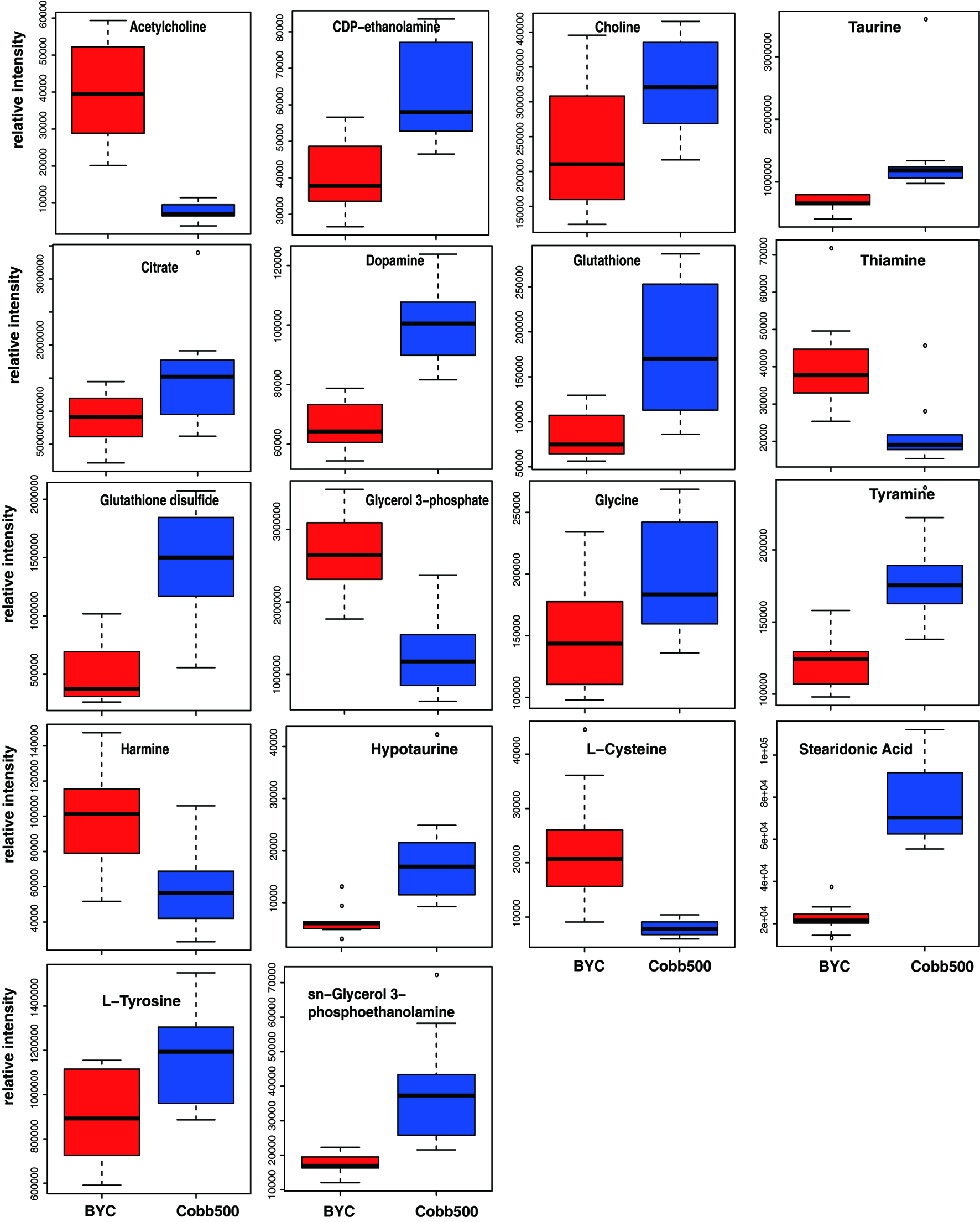

Supplement: Supplementary file 2 — Figure S2 [file FSN3-10-487-s003.tif]
